# Implementation of home‐based telemonitoring and teleconsultations for obstetrics: A cross‐sectional survey of current practice in the United Kingdom

**DOI:** 10.1002/ijgo.71205

**Published:** 2026-07-09

**Authors:** Jack Le Vance, Leo Gurney, Victoria Hodgetts Morton, R. Katie Morris

**Affiliations:** ^1^ Department of Applied Health Sciences, School of Health Sciences, College of Medicine and Health University of Birmingham Birmingham UK; ^2^ Birmingham Women's and Children's NHS Foundation Trust Birmingham UK

**Keywords:** digital technology, fetal, maternal, obstetrics, remote monitoring, teleconsultation, telemedicine

## Abstract

**Objective:**

The requirement for regular maternal–fetal monitoring and clinical consultations has increased in recent years, placing a strain on obstetric services. In recent years, technology advancements have enabled the development of remote monitoring and teleconsultation strategies, aiming to provide care in the home setting. This cross‐sectional survey seeks to understand the current state of home‐based monitoring and teleconsultations across the United Kingdom (UK).

**Methods:**

A web‐based survey was distributed to an obstetric representative in all UK National Healthcare Service (NHS) trusts. Survey questions consisted of multiple‐choice and open questions surrounding four domains: (1) demographics, (2) home‐based antenatal monitoring, (3) obstetric teleconsultations, and (4) advantages and disadvantages of home‐based monitoring and teleconsultations.

**Results:**

In total, 76 out of 145 (52.4%; 95% confidence interval [CI], 44.0–60.8 response rate) UK NHS trusts completed the survey. Any form of home monitoring and obstetric teleconsultations were provided in 61.8% (95% CI, 50.0–72.8) (47/76) and 68.4% (95% CI, 56.7–78.6) (52/76) of UK trusts, respectively. Secondary analysis demonstrated that remote monitoring was significantly more likely to be used in teaching versus non‐teaching NHS trusts (*P =* 0.03). Blood pressure monitoring (45/47, 95.8%; 95% CI, 85.5–99.5) and chronic hypertension (41/47, 87.2%; 95% CI, 74.3–95.2) were the most common device and condition to use home monitoring, respectively. Teleconsultations were utilized most frequently for patients complicated with gestational diabetes (34/52, 65.4%; 95% CI, 50.9–78.0) and delivered via a telephone call (51/52, 98.1%; 95% CI, 89.7–100). High cost and lack of clinical support were cited as the most common implementation barriers by respondents not currently using these strategies. Patient convenience (42/76, 55.3%; 95% CI, 43.4–66.7 and 44/76, 57.9%; 95% CI, 46.0–69.1) and reduced hospital burden (38/76, 50%; 95% CI, 38.3–61.7 and 22/76, 28.9%; 95% CI, 19.1–40.5) were common advantages for home monitoring and teleconsultations, respectively. Compliance issues (23/76, 30.3%; 95% CI, 20.2–41.9 and 15/76, 19.7%; 95% CI, 11.5–30.5) and digital resource requirements (15/76, 19.7%; 95% CI, 11.5–30.5, and 18/76, 23.7%; 95% CI, 14.7–34.8) were frequently cited difficulties for both home monitoring and teleconsultations, respectively.

**Conclusion:**

This study describes the current state of remote monitoring and obstetric teleconsultation implementation across the UK, with survey respondents primarily derived from English maternity services. National frameworks are emerging and are vital to inform future digital standardization.

## 1 INTRODUCTION

Obstetric care frequently encompasses maternal–fetal monitoring, such as urine testing, blood pressure, and cardiotocography combined with regular clinical consultations.^1^ The frequency of monitoring and appointments varies based on whether the patient is classified as low‐ or high‐risk, with the latter frequently receiving additional outpatient care. Over recent years, the proportion of pregnant women classifying as high risk has consistently risen, primarily due to an aging maternal population, improved clinical detection of high‐risk conditions and modifications to clinical guidelines.^2^, ^3^ Consequently, the requirements for maternal–fetal monitoring and clinical consultations have increased, placing a strain on the obstetric service to accommodate this increased demand.

In recent years, advancements in technology have enabled the development of remote monitoring strategies and virtual teleconsultations, fostering the digitization of obstetric care.^3^ The recent pandemic has had a major influence on the integration of digital/remote care, as hospitals were required to consider alternative avenues for maintaining service provision.^4^, ^5^ Published randomized controlled trial data utilizing home‐based monitoring and teleconsultation services have demonstrated that obstetric telehealth is a feasible, acceptable, and potentially an economic endeavor, while demonstrating similar clinical outcomes to routine care.^6^, ^7^, ^8^, ^9^ Vitally, digital development is a key pillar of the recent National Health Services' (NHS) Long Term Plan for England, while enabling digitally linked services to enhance safe and personalized maternity care is further endorsed in the NHS England maternity care policy, ‘Better Births’.^10^, ^11^

Consequently, in recent years, the advent of ‘digital midwives’ and digital leads in obstetrics have been developed on a local, regional and national level to help support the digital transformation in maternity.^12^ While promising, there remains a current lack of consistency in obstetric telehealth integration in maternity services across the United Kingdom (UK), with many units taking different approaches. A lack of clear guidance for telehealth usage in clinical guidelines means there remains a disparity in digital maturity across maternity units. Currently, there is a paucity of existing literature detailing the digital landscape across UK maternity services. Therefore, this cross‐sectional survey aims to shed light on the current implementation of obstetric home‐based and teleconsultation services across the nation. This includes the type of telehealth interventions used, the indications for their usage, and the local standards in place to ensure effective utilization.

## 2 MATERIALS AND METHODS

This cross‐sectional nationwide study was conducted by distributing a web‐based survey encompassing aspects relating to home‐based monitoring and teleconsultations to an obstetric representative in all NHS trusts, across all four devolved nations of the UK. In the UK, NHS trusts are healthcare organizations responsible for delivering services across one or more hospital sites. Within maternity care, NHS trusts might include a combination of district general hospitals providing consultant‐led obstetric services, tertiary referral centers offering specialist and high‐risk maternal–fetal medicine care, and quaternary centers delivering highly specialized services for the most complex conditions. Many NHS trusts are also affiliated with universities/medical schools as teaching trusts, supporting education and clinical research improvement. As a result, the organizational structure of NHS trusts reflects a tiered and integrated model of maternity care delivery, rather than a single‐site hospital‐based system. All NHS trusts consisting of a maternity department with staffed obstetricians were invited to participate in the survey. A list of trusts was acquired using the NHS maternity finder website and an obstetric consultant representative was sought using a variety of established networks, such as labor ward leads and via the local development leads list published on the Royal College of Obstetricians and Gynecologists website.^13^, ^14^ Where possible, obstetric consultants with leadership or technology roles (clinical directors, head of obstetrics, digital leads) were primarily contacted due to their likely increased awareness and involvement in local telehealth integration. A survey link was sent via email in September 2025, and representatives received a maximum of two additional email reminders to complete the survey. If no response was received, then a second obstetric representative was sought, and the above process was repeated. Informed consent was obtained by voluntary completion of the survey. A £25 voucher was provided to each respondent who completed the survey.

The survey consisted of 97 individual questions; however, the total number required for completion was dictated by the respondents' answers. The number of questions requiring completion ranged from 16 to 52 depending on the provision of home‐based monitoring and teleconsultation services within the respective NHS trust. The survey was developed through a collaborative process in the research team, which included a professor of obstetrics, two associate professors of obstetrics, and a clinical research fellow, all with extensive research and clinical experience in obstetric telemonitoring/home‐based interventions while currently caring for pregnant women. The development of questions was additionally informed by prior published non‐UK obstetric telemonitoring survey alongside established frameworks for survey research.^15^, ^16^ Patients and members from the community were not involved in the design, conduct, reporting, or dissemination of the study.

Survey questions consisted of multiple‐choice questions and open questions surrounding four main domains: (1) demographic questions, (2) home‐based antenatal monitoring, (3) obstetric teleconsultations, and (4) advantages and disadvantages of home‐based monitoring and teleconsultations (Supplementary File S1). Demographic data included the name of the NHS trust, the average births per year, the level of medical care provided by the trust and whether it is teaching affiliated. Home‐based and teleconsultation domains included a variety of questions relating to technological, governance and clinical provision. Responses to the open questions regarding the advantages and disadvantages of home‐based monitoring and teleconsultations were read by the authors and grouped into categories following consensus. Respondents were able to select multiple answers for several questions within the survey.

The survey's primary objective was to seek a deep understanding of the current state of home‐based monitoring and teleconsultations across the UK, aiming to gather responses from NHS trusts ranging in capacity and geographical location. Home‐based maternal–fetal monitoring was defined as pregnancy monitoring conducted in the home setting by the pregnant women during the antenatal period. Teleconsultations were defined as virtual consultations between the provider and another person, which could be the patient or an alternative healthcare professional, or both. Secondary analyses were conducted to examine the association between the current implementation of remote monitoring and teleconsultations and hospital characteristics, including hospital category (district, tertiary, and quaternary), hospital teaching status, and size (defined by mean annual birth rate). Descriptive statistics were presented in the form of tables and figures. Frequency and percentages with 95% confidence intervals (CIs) were used for categorical variables. 95% CIs were calculated using the Clopper–Pearson exact method. Differences between two groups was compared using the Mann–Whitney *U*‐test for continuous non‐parametric variables and either the *χ* ^2^‐ or Fisher exact test for categorical variables, depending on the event count number. This study is in accordance with the CROSS‐reporting checklist for survey studies (Table S1).^17^ The study was approved as part of a wider multi‐work package study by the London – Westminster Research Ethics Committee (REC: 25/LO/0255) on April 15, 2025, and the Health Research Authority (IRAS351514).

## 3 RESULTS

In total, 76 out of 145 NHS trusts across all four devolved nations in the UK completed the survey, demonstrating a 52.4% (95% CI, 44.0–60.8) response rate. Survey respondents, primarily derived from England (*n =* 60, 78.9%; 95% CI, 68.1–87.5), were district‐level NHS trusts (*n =* 54, 71.1%; 95% CI, 59.5–80.9) and were teaching affiliated (*n =* 64, 84.2%; 95% CI, 74.0–91.6). The median (range) annual birth rate was 4300 (150–16 200). Complete demographic data can be seen in Table 1. A distribution of nationwide survey respondents, delineated by county, can be seen in Figure 1.

**TABLE 1:** Demographic data of all survey respondents.

	Frequency	Percent (%)	95% CI
Nation			
England	60/76	78.9	68.1–87.5
Scotland	10/76	13.2	6.5–22.9
Wales	3/76	3.9	0.8–11.1
Northern Ireland	3/76	3.9	0.8–11.1
Births per year			
< 1000	1/76	1.3	0.0–7.1
1000–2000	5/76	6.6	2.2–14.7
2001–3000	15/76	19.7	11.5–30.5
3001–4000	16/76	21.1	12.5–31.9
4001–5000	13/76	17.1	9.4–27.5
5001–6000	17/76	22.4	13.6–33.4
6001–7001	4/76	5.3	1.5–12.9
7001–8000	2/76	2.6	0.3–9.2
≥ 8001	3/76	3.9	0.8–11.1
Level of maternity care			
District	54/76	71.1	59.5–80.9
Tertiary	21/76	27.6	18.0–39.1
Quaternary	1/76	1.3	0.0–7.1
Teaching hospital			
Yes	64/76	84.2	74.0–91.6
No	12/76	15.8	8.4–26.0
Provision of home monitoring			
Yes	47/76	61.8	50.0–72.8
No	27/76	35.5	24.9–47.3
No, but have in the past	2/76	2.6	0.3–9.2
Births per year in maternity units using home monitoring (mean [SD])	4744 ± 2322		
Provision of teleconsultations			
Yes	52/76	68.4	56.7–78.6
No	12/76	15.8	8.4–26.0
No, but have in the past	12/76	15.8	8.4–26.0
Births per year in maternity units using teleconsultations (mean (SD))	4683 ± 2357		

Abbreviations: CI, confidence interval; SD, standard deviation.

**FIGURE 1:**
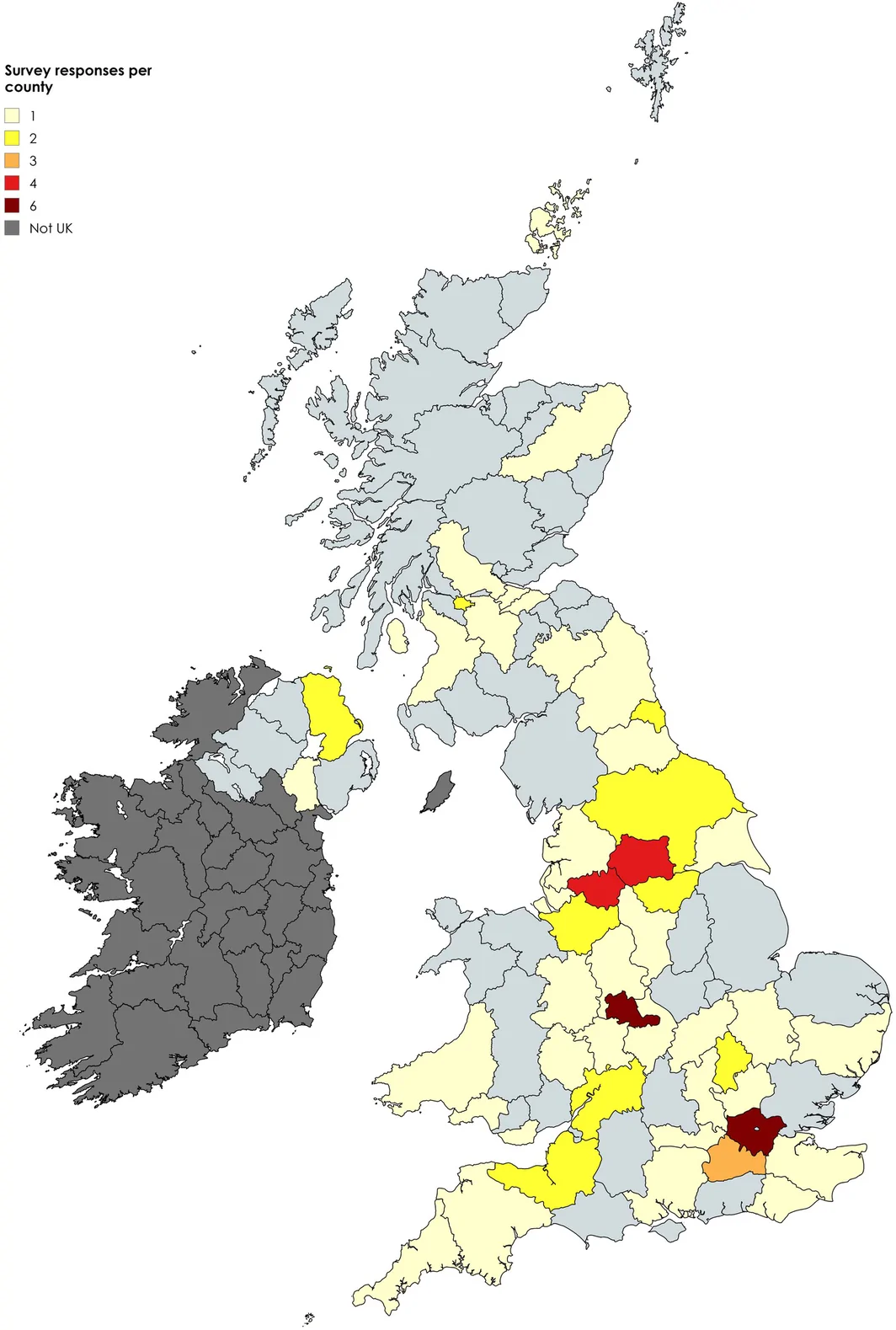
Distribution of survey respondents across the UK by county. Produced with permission from www.mapchart.net/world.html.

As of 2025, some form of home monitoring and obstetric teleconsultations were provided in 61.8% (95% CI, 50.0–72.8) and 68.4% (95% CI, 56.7–78.6) of UK NHS trusts respectively.

### 3.1 Secondary analysis of service adoption

Secondary analysis assessed associations between service adoption and trust characteristics. Usage of remote monitoring and obstetric teleconsultations were not significantly associated with the size of the trust (dictated by mean annual birth rate) (4744 ± 2322 versus 4055 ± 1740, *P =* 0.22 and 4683 ± 2357 versus 4068 ± 1551, *P =* 0.18 respectively). Remote monitoring was significantly more likely to be used in teaching‐affiliated NHS trusts (*P =* 0.03), albeit no difference was seen in district versus tertiary/quaternary NHS trusts (*P =* 0.60). In contrast, implementation of teleconsultations was not statistically different in teaching versus non‐teaching affiliated trusts (*P =* 1.00) and district versus tertiary/quaternary trusts (*P =* 0.18). There were no statistically significant differences in demographics characteristics between respondents and non‐respondents of the survey (Table S2).

## 4 HOME MONITORING

### 4.1 Current users of home monitoring

#### 4.1.1 Adoption and temporal trends

Home monitoring was in use in 47/76 NHS trusts (61.8%; 95% CI, 50.0–72.8). For respondents that specifically detailed the earliest year when home monitoring was implemented (*n =* 39), the median year was 2019 (2005–2024). A sharp increase in the provision of home monitoring was seen in 2020, coinciding with the COVID pandemic (Figure 2). When asked, 87.2% (95% CI, 74.3–95.2) of respondents (*n =* 41) had noticed an increase home monitoring usage following the pandemic.

**FIGURE 2:**
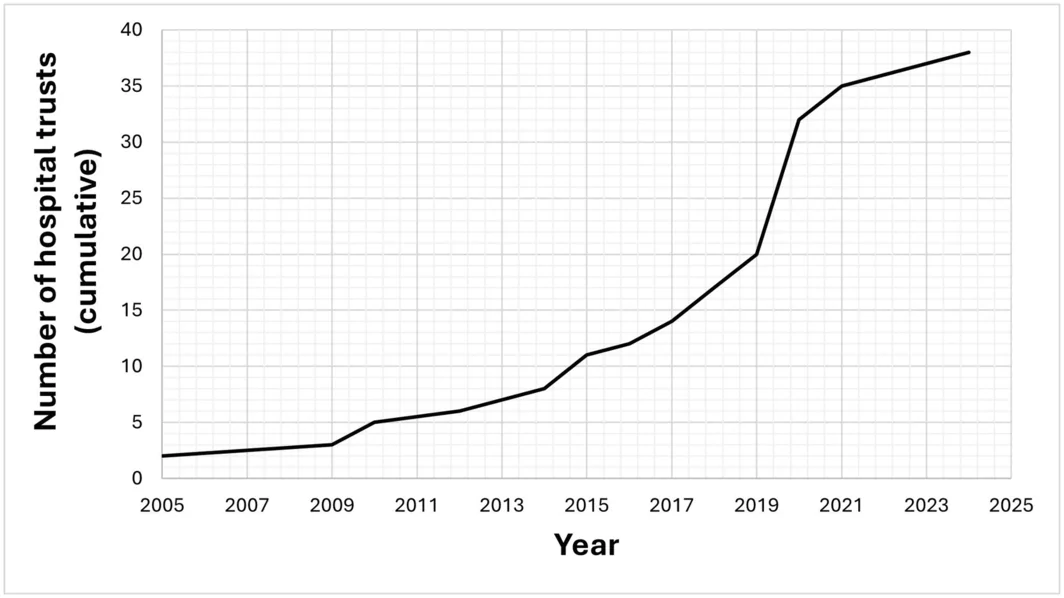
Temporal trends in the implementation of home monitoring services between 2005 and 2024, shown as the cumulative number of survey respondent hospital trusts.

#### 4.1.2 Models of care and patient selection

Home monitoring was predominately provided to only high‐risk obstetric patients (*n =* 25, 53.2%; 95% CI, 38.1–67.9), followed by both high‐ and low‐risk (*n =* 20, 42.6%; 95% CI, 28.3–57.8) and then only low‐risk patients (*n =* 2, 4.3%; 95% CI, 0.5–14.5). Blood pressure monitoring (*n =* 45, 95.8%; 95% CI, 85.5–99.5) and glucose monitoring (*n =* 41, 87.2%; 95% CI, 74.3–95.2) were the most common types of home monitoring, and chronic hypertension (*n =* 41, 87.2%; 95% CI, 74.3–95.2) and gestational diabetes (85.1%; 95% CI 71.7–93.8) were the most common conditions to utilize home monitoring (Table 2).

**TABLE 2:** Results of survey respondents in maternity trusts which are current users of home obstetric monitoring.

	Frequency	Percent (%)	95% CI
Type of home monitoring^a^			
Blood pressure monitoring	45/47	95.8	85.5–99.5
Glucose monitoring	41/47	87.2	74.3–95.2
Temperature measurement	20/47	42.6	28.3–57.8
Urine testing	14/47	29.8	17.3–44.9
Maternal heart rate measurement	2/47	4.3	0.5–14.5
High‐risk obstetric conditions using home monitoring^a^			
Cardiovascular			
Chronic hypertension	41/47	87.2	74.3–95.2
Gestational hypertension	37/47	78.7	64.3–89.3
Pre‐eclampsia	28/47	59.6	44.3–73.6
Endocrine and metabolic			
Gestational diabetes	40/47	85.1	71.7–93.8
Pre‐existing diabetes	3/47	6.4	1.3–17.5
Obstetric complications – membrane rupture			
Preterm prelabor rupture of membranes	16/47	34.0	20.9–49.3
Prolonged rupture of membranes at term	13/47	27.7	15.6–42.6
Fetal conditions			
Fetal growth restriction	3/47	6.4	1.3–17.5
Reduced fetal movements	2/47	4.3	0.5–14.5
Pregnancy‐related risk factors			
Poor obstetric history	3/47	6.4	1.3–17.5
Multiple pregnancy	1/47	2.1	0.1–11.3
Psychosocial			
Social/psychological reasons	1/47	2.1	0.1–11.3
Healthcare professional reviewing home monitoring data^a^			
Specialist midwife	33/47	70.2	55.1–82.7
Midwife	31/47	66.0	50.7–79.1
Obstetric consultant	22/47	46.8	32.1–61.9
Doctor – non consultant	14/47	29.8	17.3–44.9
Specialist nurse	4/47	8.5	2.4–20.4
Healthcare assistant	1/47	2.1	0.1–11.3
Method of communication to feedback results to patient^a^			
Phone call	37/47	78.7	64.3–89.3
Text message	14/47	29.8	17.3–44.9
Patient portals	14/47	29.8	17.3–44.9
Face to face	6/47	12.8	4.8–25.7
Email	5/47	10.6	3.5–23.1
Barriers for home monitoring^a^			
Non‐compliance issues	38/47	80.9	66.7–90.9
Language barriers	35/47	74.5	59.7–86.1
Patient too complex	9/47	19.1	9.1–33.3
Distance from home to hospital	5/47	10.6	3.5–23.1
Decided on an individual basis	5/47	10.6	3.5–23.1
Social/psychological concerns	4/47	8.5	2.4–20.4
None	3/47	6.4	1.3–17.5
BMI	2/47	4.3	0.5–14.5
Needlephobia	1/47	2.1	0.1–11.3
Minimum gestational age for home monitoring			
First trimester	3/47	6.4	1.3–17.5
Second trimester	4/47	8.5	2.4–20.4
Third trimester	2/47	4.3	0.5–14.5
Unsure	2/47	4.3	0.5–14.5
No minimum gestation	36/47	76.6	62.0–87.7
Local guideline for home monitoring			
Yes	40/47	85.1	71.7–93.8
No	3/47	6.4	1.3–17.5
Not sure	4/47	8.5	2.4–20.4
Patient safety evaluation prior to using home monitoring			
Yes	26/47	55.3	40.1–69.8
No	4/47	8.5	2.4–20.4
Not sure	17/47	36.2	22.7–51.5
Assessment of home monitoring on patient experiences			
Yes	13/47	27.7	15.6–42.6
No	19/47	40.4	26.4–55.7
Not sure	15/47	31.9	19.1–47.1
Assessment of home monitoring on clinical outcomes			
Yes	19/47	40.4	26.4–55.7
No	21/47	44.7	30.2–59.9
Not sure	7/47	14.9	6.2–28.3
Assessment of home monitoring on healthcare service outcomes			
Yes	13/47	27.7	15.6–42.6
No	25/47	53.2	38.1–67.9
Not sure	9/47	19.1	9.1–33.3

Abbreviations: BMI, body mass index; CI, confidence interval.

^a^ Respondents were able to select multiple answers.

#### 4.1.3 Service delivery and governance

Most frequently, home monitoring data was evaluated by a specialist midwife (*n =* 33, 70.2%; 95% CI, 55.1–82.7), and results were communicated back to the patient remotely via a phone call (*n =* 37, 78.7%; 95% CI, 64.3–89.3). Most trusts reported having a local guideline for home monitoring (85.1%; 95% CI, 71.7–93.8) of trusts (*n =* 40). However, a patient safety evaluation prior to implementation had been conducted in just 55.3% (95% CI, 40.1–69.8) of NHS trusts (*n =* 26). According to respondents, the impact of home monitoring on clinical outcomes, healthcare usage outcomes, and patient experience was assessed in 40.4% (95% CI, 26.4–55.7), 27.7% (95% CI, 15.6–42.6), and 27.7% (95% CI, 15.6–42.6) of trusts, respectively. A full description of results can be seen in Table 2.

### 4.2 Non‐users of home monitoring

In 27 of the 76 NHS trusts (35.5%; 95% CI, 24.9–47.3), home monitoring had never been implemented. The most common reported barriers were the lack of clinical team support (*n =* 11, 40.7%; 95% CI, 22.4–61.2) and the cost (*n =* 11, 40.7%;95% CI, 22.4–61.2). Only 33.3% (95% CI, 16.5–54.0) of trusts were considering the future implementation of home monitoring, most commonly using home blood pressure monitoring (55.6%; 95% CI, 21.2–86.3) (Table 3).

**TABLE 3:** Results of survey respondents in maternity trusts which are current users of home obstetric monitoring.

	Frequency	Percent (%)	95% CI
Reason for not providing home monitoring^a^			
Lack of clinical support	11/27	40.7	22.4–61.2
Cost too high	11/27	40.7	22.4–61.2
Lack of clinical capacity	6/27	22.2	8.6–42.3
Never been considered	4/27	14.8	4.2–33.7
Lack of patient interest	3/27	11.1	2.4–29.2
Not enough patients	2/27	7.4	0.9–24.3
Safety concerns	1/27	3.7	0.1–19.0
Lack of evidence for its use	1/27	3.7	0.1–19.0
Considerations of future home monitoring			
Yes	9/27	33.3	16.5–54.0
No	18/27	66.7	46.0–83.5

Abbreviation: CI, confidence interval.

^a^ Respondents were able to select multiple answers.

### 4.3 Previous users of home monitoring services

Two NHS trusts (2.6%; 95% CI, 0.3–9.2) previously provided home monitoring but have since then discontinued. Forms of home monitoring included blood pressure monitoring (*n =* 2, 100%; 95% CI, 15.8–100), glucose monitoring (*n =* 2, 100%; 95% CI, 15.8–100), fetal electrocardiography (*n =* 1, 50.0%; 95% CI, 1.3–98.7), and temperature measurement (*n =* 1, 50.0%; 95% CI, 1.3–98.7). Home monitoring was discontinued due to cost (*n =* 1, 50.0%; 95% CI, 1.3–98.7), lack of clinical team support (*n =* 1, 50.0%; 95% CI, 1.3–98.7), and a lack of patient interest (*n =* 1, 50.0%; 95% CI, 1.3–98.7). One NHS trust is planning to recommence home monitoring in the future. Full details can be found in Table S3.

## 5 TELECONSULTATIONS

### 5.1 Current users of teleconsultations

#### 5.1.1 Adoption and temporal trends

Fifty‐two (68.4%; 95% CI, 56.7–78.6) NHS trusts currently provide obstetric teleconsultations. Similar to home monitoring, uptake sharply rose in 2020 during the COVID pandemic (Figure 3), with 90.4% (95% CI, 79.0–96.8) of respondents reporting increased use. For respondents that specifically detailed the earliest year when teleconsultations were implemented (*n =* 44, 84.6%; 95% CI, 71.9–93.1), the median was 2020 2020 (2011–2022).

**FIGURE 3:**
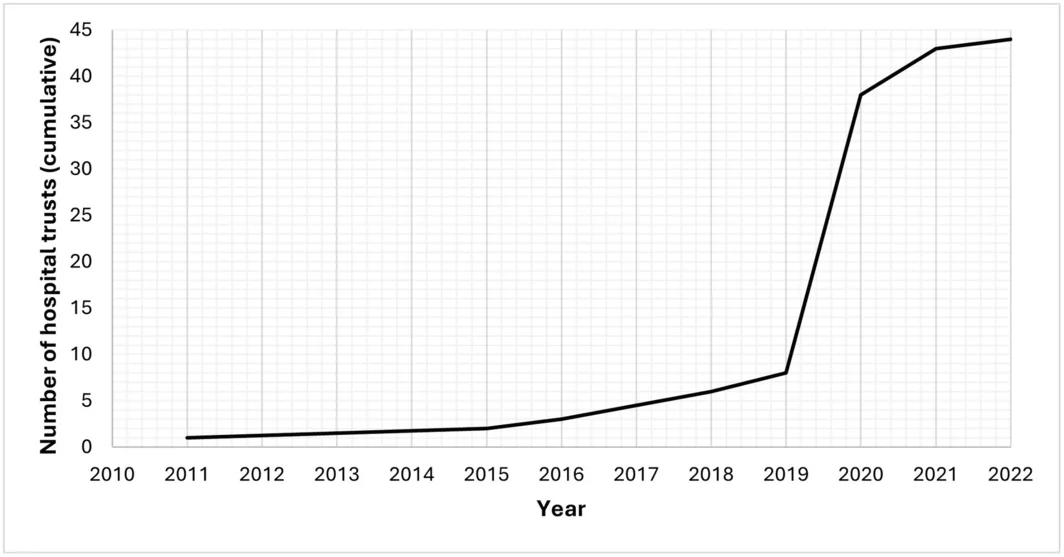
Temporal trends in the implementation of teleconsultation services between 2011 and 2022, shown as the cumulative number of survey respondent hospital trusts.

#### 5.1.2 Models of care and patient selection

Teleconsultations were most commonly conducted during the antenatal period (*n =* 41, 78.8%; 95% CI, 65.3–88.9) and predominately provided to both high‐ and low‐risk obstetric patients (*n =* 37, 71.2%; 95% CI, 56.9–82.9), followed by only high‐risk (*n =* 10, 19.2%; 95% CI, 9.6–32.5) and then only low‐risk patients (*n =* 5, 9.6%; 95% CI, 3.2–21.0). The most common high‐risk condition to engage with teleconsultations was those complicated with gestational diabetes (*n =* 34, 65.4%; 95% CI, 50.9–78.0). Teleconsultations were mostly conducted by a consultant (*n =* 48, 92.3%; 95% CI, 81.5–97.9) and delivered via a telephone call (*n =* 51, 98.1%; 95% CI, 89.7–100) (Table 4).

**TABLE 4:** Results of survey respondents in maternity trusts which are current users of obstetric teleconsultations.

	Frequency	Percent (%)	95% CI
Type of teleconsultations^a^			
Antenatal clinic appointments	44/52	84.6	71.9–93.1
Discuss home monitoring results	26/52	50.0	35.8–64.2
Postnatal clinic appointments	24/52	46.2	32.2–60.5
Pre–pregnancy clinic appointments	23/52	44.2	30.5–58.7
To discuss with other specialists	21/52	40.4	27.0–54.9
Teleultrasound services	4/52	7.7	2.1–18.5
High–risk obstetric conditions using teleconsultations^a^			
Cardiovascular			
Gestational hypertension	20/52	38.5	25.3–53.0
Chronic hypertension	18/52	34.6	22.0–49.1
Pre‐eclampsia	12/52	23.1	12.5–36.8
Endocrine and metabolic			
Gestational diabetes	34/52	65.4	50.9–78.0
Hepatobiliary			
Obstetric cholestasis	11/52	21.1	11.1–34.7
Obstetric complications – membrane rupture			
Preterm prelabor rupture of membranes	11/52	21.2	11.1–34.7
Prolonged prelabor rupture of membranes	7/52	13.5	5.6–25.8
Fetal conditions			
Fetal growth restriction	10/52	19.2	9.6–32.5
Fetal anomaly	8/52	15.4	6.9–28.1
Reduced fetal movements	6/52	11.5	4.4–23.4
Amniotic fluid disorders			
Polyhydramnios	7/52	13.5	5.6–25.8
Oligohydramnios	4/52	7.7	2.1–18.5
Pregnancy‐related risk factors			
Poor obstetric history	17/52	32.7	20.3–47.1
Multiple pregnancy	7/52	13.5	5.6–25.8
Previous caesarean section	2/52	3.9	0.5–13.2
Maternal medical conditions			
Complex maternal medical conditions	7/52	13.5	5.6–25.8
Psychosocial			
Social/psychological reasons	15/52	28.9	17.1–43.1
Healthcare professional conducting teleconsultations^a^			
Obstetric consultant	48/52	92.3	81.5–97.9
Doctor – non consultant	36/52	69.2	54.9–81.3
Specialist midwife	35/52	67.3	52.9–79.7
Midwife	31/52	59.6	45.1–73.0
Non obstetric doctor	1/52	1.9	0.0–10.3
Method of communication for teleconsultations^a^			
Phone call	51/52	98.1	89.7–100
Video call	20/52	38.5	25.3–53.0
Text message	8/52	15.4	6.9–28.1
Patient portals	8/52	15.4	6.9–28.1
Barriers for teleconsultations^a^			
Language barriers	33/52	63.5	49.0–76.4
Non–compliance issues	29/52	55.8	41.3–69.5
Social/psychological concerns	10/52	19.2	9.6–32.5
Distance from home to hospital	8/52	15.4	6.9–28.1
Patient too complex	4/52	7.7	2.1–18.5
Decided on an individual basis	3/52	5.8	1.2–15.9
Lack of patient technology infrastructure	2/52	3.8	0.5–13.2
Digital literacy	1/52	1.9	0.0–10.3
None	1/52	1.9	0.0–10.3
Minimum gestational age for teleconsultations			
First trimester	1/52	1.9	0.0–10.3
Second trimester	0/52	0	0.0–6.8
Third trimester	1/52	1.9	0.0–10.3
Unsure	3/52	5.8	1.2–15.9
No minimum gestation	47/52	90.4	79.0–96.8
Local guideline for teleconsultations			
Yes	14/52	26.9	15.6–41.0
No	29/52	55.8	41.3–69.5
Not sure	9/52	17.3	8.2–30.3
Patient safety evaluation prior to using teleconsultations			
Yes	11/52	21.2	11.1–34.7
No	23/52	44.2	30.5–58.7
Not sure	18/52	34.6	22.0–49.1
Assessment of teleconsultations on patient experiences			
Yes	8/52	15.4	6.9–28.1
No	28/52	53.8	39.5–67.8
Not sure	16/52	30.8	18.7–45.1
Assessment of teleconsultations on clinical outcomes			
Yes	8/52	15.4	6.9–28.1
No	36/52	69.2	54.9–81.3
Not sure	8/52	15.4	6.9–28.1
Assessment of teleconsultations on healthcare service outcomes			
Yes	11/52	21.2	11.1–34.7
No	36/52	69.2	54.9–81.3
Not sure	5/52	9.6	3.2–21.0

Abbreviation: CI, confidence interval.

^a^ Respondents were able to select multiple answers.

#### 5.1.3 Service delivery and governance

Respondents reported that a local guideline for teleconsultations was in place in 26.9% (95% CI 15.6–41.0) of NHS trusts (*n =* 14), and a patient safety evaluation prior to implementation was conducted in just 21.2% (95% CI, 11.1–34.7) of trusts (*n =* 11). According to respondents, the impact of teleconsultations on clinical outcomes, healthcare usage outcomes, and patient experience was assessed in 15.4% (95% CI, 6.9–28.1), 21.2% (95% CI, 11.1–34.7) and 15.4% (95% CI, 6.9–28.1) of NHS trusts, respectively. A full description of results can be seen in Table 4.

#### 5.1.4 Non‐users of teleconsultations

Teleconsultations had never been provided in 12 of the 76 NHS trusts (15.8%; 95% CI, 8.4–26.0) responding to this survey. The most common reasons for not implementing teleconsultations are due the cost being too high (*n =* 5, 41.7%; 95% CI, 15.2–72.3) and lack of clinical support (*n =* 4, 33.3%; 95% CI, 9.9–65.1). Only 25% (95% CI, 5.5–57.2) of NHS trusts were considering the future integration of teleconsultations (Table 5).

**TABLE 5:** Results of survey respondents in maternity trusts which are non‐users of obstetric teleconsultations.

	Frequency	Percent (%)	95% CI
Reason for not providing teleconsultations^a^			
Cost too high	5/12	41.7	15.2–72.3
Lack of clinical support	4/12	33.3	9.9–65.1
Lack of clinical capacity	2/12	16.7	2.1–48.4
Lack of patient interest	2/12	16.7	2.1–48.4
Not enough patients	2/12	16.7	2.1–48.4
Lack of equipment	1/12	8.3	0.2–38.5
Preference with face‐to‐face	1/12	8.3	0.2–38.5
Considerations of future teleconsultations			
Yes	3/12	25.0	5.5–57.2
No	9/12	75.0	42.8–94.5

Abbreviation: CI, confidence interval.

^a^ Respondents were able to select multiple answers.

#### 5.1.5 Previous users of teleconsultation services

Twelve NHS trusts (15.8%; 95% CI, 8.4–26.0) previously provided obstetric teleconsultations but have since then discontinued. Ten trusts (83.3%; 95% CI, 51.6–97.9) employed teleconsultations only during the COVID pandemic. The most common reason for discontinuation was due to lack of clinical capacity (*n =* 6, 50%; 95% CI, 21.1–78.9). The most common high‐risk conditions to engage with teleconsultations were patients complicated by gestational or chronic hypertension (*n =* 10, 83.3%; 95% CI, 51.6–97.9). Two NHS trusts (16.7%; 95% CI, 2.1–48.4) are considering recommencing teleconsultations in the near future. A full description of results can be seen in Table 6.

**TABLE 6:** Results of survey respondents in maternity trusts which are previous users of obstetric teleconsultations.

	Frequency	Percent (%)	95% CI
Reason for discontinuation^a^			
Lack of clinical capacity	6/12	50.0	21.1–78.9
Lack of clinical support	3/12	25.0	5.5–57.2
Lack of patient interest	3/12	25.0	5.5–57.2
Adverse outcomes	2/12	16.7	2.1–48.4
Not enough patients	1/12	8.3	0.2–38.5
Technological insufficiencies	1/12	8.3	0.2–38.5
Cost too high	0/12	0	0.0–26.5
Years of teleconsultation implementation			
2020–2022	6/12	50.0	21.1–78.9
2020–2021	3/12	25.0	5.5–57.2
2021	1/12	8.3	0.2–38.5
2019–2020	1/12	8.3	0.2–38.5
2016	1/12	8.3	0.2–38.5
Type of teleconsultations^a^			
Antenatal clinic appointments	10/12	83.3	51.6–97.9
Pre‐pregnancy clinic appointments	5/12	41.7	15.2–72.3
Discuss home monitoring results	2/12	16.7	2.1–48.4
Postnatal clinic appointments	2/12	16.7	2.1–48.4
To discuss with other specialists	1/12	8.3	0.2–38.5
Teleultrasound services	0/12	0	0.0–26.5
High‐risk obstetric conditions using teleconsultations^a^			
Cardiovascular			
Gestational hypertension	10/12	83.3	51.6–97.9
Chronic hypertension	10/12	83.3	51.6–97.9
Pre‐eclampsia	7/12	58.3	27.7–84.8
Endocrine and metabolic			
Gestational diabetes	8/12	66.7	34.9–90.1
Pre‐existing diabetes	1/12	8.3	0.2–38.5
Hepatobiliary			
Obstetric cholestasis	6/12	50.0	21.1–78.9
Obstetric complications – membrane rupture			
Preterm prelabor rupture of membranes	3/12	25.0	5.5–57.2
Prolonged prelabor rupture of membranes	2/12	16.7	2.1–48.4
Fetal conditions			
Fetal growth restriction	6/12	50.0	21.1–78.9
Fetal anomaly	3/12	25.0	5.5–57.2
Reduced fetal movements	3/12	25.0	5.5–57.2
Amniotic fluid disorders			
Polyhydramnios	4/12	33.3	9.9–65.1
Oligohydramnios	3/12	25.0	5.5–57.2
Pregnancy‐related risk factors			
Poor obstetric history	7/12	58.3	27.7–84.8
Multiple pregnancy	4/12	33.3	9.9–65.1
Psychosocial			
Social/psychological reasons	5/12	58.3	15.2–72.3
Healthcare professional conducting the teleconsultations^a^			
Obstetric consultant	12/12	100.0	73.5–100
Doctor – non consultant	7/12	58.3	27.7–84.8
Specialist midwife	6/12	50.0	21.1–78.9
Midwife	4/12	33.3	9.9–65.1
Method of communication for teleconsultations^a^			
Phone call	11/12	91.7	61.5–99.8
Video call	5/12	41.7	15.2–72.3
Patient portals	2/12	16.7	2.1–48.4
Text message	1/12	8.3	0.2–38.5
Barriers for teleconsultations^a^			
Language barriers	10/12	83.3	51.6–97.9
Non‐compliance issues	9/12	75.0	42.8–94.5
Patient too complex	9/12	75.0	42.8–94.5
Social/psychological concerns	1/12	8.3	0.2–38.5
Distance from home to hospital	1/12	8.3	0.2–38.5
None	1/12	8.3	0.2–38.5
Minimum gestational age for teleconsultations			
First trimester	3/12	25.0	5.5–57.2
Second trimester	0/12	0.0	0.0–26.5
Third trimester	0/12	0.0	0.0–26.5
Unsure	0/12	0.0	0.0–26.5
No minimum gestation	9/12	75.0	42.8–94.5
Local guideline for teleconsultations			
Yes	4/12	33.3	9.9–65.1
No	8/12	66.7	34.9–90.1
Not sure	0/12	0.0	0.0–26.5
Patient safety evaluation prior to using teleconsultations			
Yes	2/12	16.7	2.1–48.4
No	6/12	50.0	21.1–78.9
Not sure	4/12	33.3	9.9–65.1
Assessment of teleconsultations on patient experiences			
Yes	2/12	16.7	2.1–48.4
No	7/12	58.3	27.7–84.8
Not sure	3/12	25.0	5.5–57.2
Assessment of teleconsultations on clinical outcomes			
Yes	1/12	8.3	0.2–38.5
No	9/12	75.0	42.8–94.5
Not sure	2/12	16.7	2.1–48.4
Assessment of teleconsultations on healthcare service outcomes			
Yes	1/12	8.3	0.2–38.5
No	10/12	83.3	51.6–97.9
Not sure	1/12	8.3	0.2–38.5

Abbreviation: CI, confidence interval.

^a^ Respondents were able to select multiple answers.

#### 5.1.6 Advantages and disadvantages of remote monitoring and teleconsultations by all survey respondents

Advantages of home monitoring and obstetric teleconsultations, as perceived by respondents, frequently related to patient convenience (*n =* 42, 55.3%; 95% CI, 43.4–66.7 and *n =* 44, 57.9%; 95% CI, 46.0–69.1) and a reduction on hospital burden (*n =* 38, 50%; 95% CI, 38.3–61.7 and *n =* 22, 28.9%; 95% CI, 19.1–40.5). Additional perceived benefits include time savings, economic benefit, and improved patient satisfaction (Table 7).

**TABLE 7:** Advantages and disadvantages of home monitoring and obstetric teleconsultations, provided by all survey respondents.

	Frequency	Percent (%)	95% CI
Advantages for home monitoring^a^			
Patient convenience	42/76	55.3	43.4–66.7
Reduced burden for the hospital	38/76	50.0	38.3–61.7
Enhancement in antenatal monitoring	13/76	17.1	9.4–27.5
Patient satisfaction	11/76	14.5	7.5–24.4
Equity in care	10/76	13.2	6.5–22.9
Saves time	7/76	9.2	3.8–18.1
Patient empowerment	6/76	7.9	3.0–16.4
Staff convenience	5/76	6.6	2.2–14.7
Economic benefit	5/76	6.6	2.2–14.7
Patient relaxation	5/76	6.6	2.2–14.7
Patient compliance	4/76	5.3	1.5–12.9
Increase workload	3/76	3.9	0.8–11.1
Environmentally sustainable	2/76	2.6	0.3–9.2
Patient autonomy	2/76	2.6	0.3–9.2
Patient reassurance	1/76	1.3	0.0–7.1
Patient engagement	1/76	1.3	0.0–7.1
Patient safety	1/76	1.3	0.0–7.1
Disadvantages for home monitoring^a^			
Compliance issues	23/76	30.3	20.2–41.9
Lack of face‐to‐face care	16/76	21.1	12.5–31.9
Digital resource	15/76	19.7	11.5–30.5
Miss additional information	13/76	17.1	9.4–27.5
Cost	12/76	15.8	8.4–26.0
Language barriers	10/76	13.2	6.5–22.9
Inaccurate data	10/76	13.2	6.5–22.9
Patient reliability	10/76	13.2	6.5–22.9
Equipment reliability	6/76	7.9	3.0–16.4
Patient technique	6/76	7.9	3.0–16.4
Lack of real–time data	5/76	6.6	2.2–14.7
Reduces rapport	5/76	6.6	2.2–14.7
Provision of staff	4/76	5.3	1.5–12.9
Governance issues	4/76	5.3	1.5–12.9
Inequalities in care	4/76	5.3	1.5–12.9
Lack of patient understanding	3/76	3.9	0.8–11.1
Increases staff time	3/76	3.9	0.8–11.1
Lack of data follow‐up	2/76	2.6	0.3–9.2
Lost equipment	2/76	2.6	0.3–9.2
Safety	1/76	1.3	0.0–7.1
Increases admin	1/76	1.3	0.0–7.1
Reduces patient preference	1/76	1.3	0.0–7.1
Requires staff training	1/76	1.3	0.0–7.1
Advantages of teleconsultations^a^			
Patient convenience	44/76	57.9	46.0–69.1
Saves time	25/76	32.9	22.5–44.6
Reduced burden for the hospital	22/76	28.9	19.1–40.5
Flexible working	14/76	18.4	10.5–29.0
Economic benefit	13/76	17.1	9.4–27.5
Technology integration/data access	7/76	9.2	3.8–18.1
Patient satisfaction	7/76	9.2	3.8–18.1
Facilitates choice	5/76	6.6	2.2–14.7
Equity in care	4/76	5.3	1.5–12.9
Environmentally sustainable	4/76	5.3	1.5–12.9
Patient compliance	2/76	2.6	0.3–9.2
Patient safety	2/76	2.6	0.3–9.2
Disadvantages of teleconsultations^a^			
Cannot examine or perform observations	28/76	36.8	26.1–48.7
Digital resource	18/76	23.7	14.7–34.8
Reduce non‐verbal communication	16/76	21.1	12.5–31.9
Compliance issues	15/76	19.7	11.5–30.5
Inability to enquire about sensitive issues	13/76	17.1	9.4–27.5
Reduces rapport	10/76	13.2	6.5–22.9
Language barriers	8/76	10.5	4.7–19.7
Lack of face‐to‐face care	8/76	10.5	4.7–19.7
No ultrasound provision	7/76	9.2	3.8–18.1
Miss additional information	7/76	9.2	3.8–18.1
Impacted by digital literacy	4/76	5.3	1.5–12.9
Inequalities in care	4/76	5.3	1.5–12.9
Increase staff time	3/76	3.9	0.8–11.1
Cost	3/76	3.9	0.8–11.1
Requires staff training	2/76	2.6	0.3–9.2
Not applicable for all women	2/76	2.6	0.3–9.2
Confidentiality concerns	1/76	1.3	0.0–7.1
Reduces provider confidence	1/76	1.3	0.0–7.1
Increases admin	1/76	1.3	0.0–7.1

Abbreviation: CI, confidence interval.

^a^ Respondents were able to select multiple answers.

Compliance issues (*n =* 23, 30.3%; 95% CI, 20.2–41.9 and *n =* 15, 19.7%; 95% CI, 11.5–30.5), digital resource requirements (*n =* 15, 19.7%; 95% CI, 11.5–30.5 and *n =* 18, 23.7%; 95% CI, 14.7–34.8), and language barriers (*n =* 10, 13.2%; 95% CI, 6.5–22.9 and *n =* 8, 10.5%; 95% CI, 4.7–19.7) were cited by respondents as common barriers for both home monitoring and obstetric teleconsultations, respectively. Teleconsultations were additionally limited by inability to perform physical examination, reduced non‐verbal communication, and reduced opportunity to explore sensitive issues. A complete list can be seen in Table 7.

## 6 DISCUSSION

### 6.1 Main findings

Results of this survey reveal the current state of home monitoring and obstetric teleconsultation implementation across the UK. Predominately survey responses arose from NHS trusts located in England (78.9%; 95% CI, 68.1–87.5). Currently, home monitoring and obstetric teleconsultations were used in 61.8% (95% CI, 50.0–72.8) and 68.4% (95% CI, 56.7–78.6) of UK NHS trusts, respectively. Blood pressure monitoring and antenatal teleconsultations were the most common types of remote monitoring and teleconsultations services employed by trusts, respectively. Non‐compliance and language barriers were typical reasons to decline offering such interventions to pregnant women. According to respondents, a local guideline for remote monitoring was more readily available than for teleconsultations (85.1%; 95% CI, 71.7–93.8 vs. 26.9%; 95% CI 15.6–41.0). However, respondents noted that the evaluation of either digital strategy on clinical outcomes, healthcare usage outcomes, or patient experience was globally poor in NHS trusts using these interventions.

For trusts not currently using either home monitoring or teleconsultations, a lack of clinical support and economic burden were common barriers to implementation. Reported advantages included patient convenience, reduced hospital burden, and optimizing time. Disadvantages included concerns regarding compliance, digital infrastructure requirements, and language barriers.

### 6.2 Interpretation

In recent years, digital integration has become one of the most promising and innovative additions to obstetric care, particularly seen due to the new availability of several remote maternal–fetal monitoring devices. The survey results demonstrate the utility of basic remote monitoring strategies, such as blood pressure, temperature measuring, and urine testing. Such devices have the potential to transform how antenatal care is provided, helping to enhance the care provided to patients and enable continuation of monitoring outside the hospital setting. Further, the recent development of more sophisticated devices has been explored, such as remote ultrasound and home cardiotocography, which have demonstrated feasibility, acceptability, non‐inferiority to conventional care, and safety.^1^, ^7^, ^9^, ^18^, ^19^, ^20^ While promising, UK large randomized controlled trial data with standardized outcome assessment and health economics analysis is currently lacking, limiting its current integration into national guideline scope and routine antenatal care models.^21^ The trajectory of medical obstetric technology research suggests that recommendations for routine usage of such approaches might take place in the near future.

As digital innovations for maternity care continue to develop, it is imperative to consider their impact on service users, particularly those experiencing health inequalities. Respondents identified lack of patient understanding, inequity in care and limited digital literacy as potential disadvantages of remote monitoring and teleconsultation initiatives. However, understanding women's experiences firsthand is equally as important to evaluate when considering implementation, an area noted to be minimally explored by survey respondents. Digital strategies might be particularly valuable for women needing to balance employment and childcare responsibilities or for those traveling long travel distances to maternity services. However, while potentially convenient, some women might value the reassurance and communication associated with face‐to‐face consultations.^22^, ^23^ Successful implementation, therefore, requires consideration of patient preferences, communication needs, and opportunities for shared decision‐making. NHS trusts must evaluate women's experiences to ensure digital services remain patient‐centered.

Presently, around 7% of households do not have home internet access and approximately 10 million adults are estimated to lack foundation‐level digital skills.^24^, ^25^ Further, in this study, few NHS trusts using remote monitoring or teleconsultation services had clear policies to support implementation, minimize digital exclusion, and evaluate these digital strategies. The absence of robust local guidelines might limit equitable access and exacerbate existing healthcare inequities. Several national initiatives have been developed to support digital inclusion. The NHS Inclusive Digital Healthcare Framework focuses on improving access to devices and connectivity, digital upskilling, and improving patient confidence using digital health approaches.^24^ Additional initiatives developed in collaboration with the NHS digital midwives leadership team and the Good Things Foundation aim to improve access to devices and connectivity while supporting digital literacy among pregnant women.^26^ Ensuring that such initiatives are translated into local policy and practice will be essential to maximize the benefits of digital health care while reducing the risk of exacerbating existing inequalities.

National integration of remote monitoring and obstetric teleconsultations requires careful consideration of ethical, regulatory, and economic factors prior to implementation. Key issues include standardization of data security, ownership, storage and protection, particularly where services are outsourced to non‐UK‐based companies. Compliance with legal and regulatory frameworks is essential to ensure patient safety and to support digital embedment across the NHS. Recent guidance from the American College of Obstetricians and Gynecologists regarding telehealth ethics highlights the growing importance of governance in digital maternity care.^27^

Economic burden is an essential consideration when widely integrating remote monitoring and obstetric teleconsultations. Reported benefits included reduced patient travel, fewer hospital attendances, and more efficient use of clinician time.^1^, ^7^, ^28^, ^29^ However, initial implementation cost, including device expenses and digital infrastructure, have been perceived as barriers for widespread usage. The overall economic impact is complex and might vary depending on local funding structures. Within the NHS tariff‐based maternity funding model, savings in travel and appointment time might not directly translate into financial benefit for the individual trust.^30^ Nevertheless, wider system‐level advantages might still arise, including more efficient use of clinical capacity and potential reallocation of resources across specialties both within maternity and outside. Importantly, remote care might also contribute to reduced environmental impact, which aligns closely with NHS net zero ambitions by 2040.^31^

Finally, the role of digital midwives varies considerably between NHS trusts contributing to differences in telehealth implementation, as evidenced from the results of this survey. Greater national guidance and standardization might help promote a more consistent adoption of digital technologies across maternity services.

### 6.3 Strengths and limitations

The main strength of this study was the diversity of survey respondents, primarily in relation to geographical spread, size of NHS trust, annual birth rate and in both teaching and nonteaching trusts. This enabled results to be informed by different models of maternity care, subsequently increasing the validity and generalizability of results. The response rate was moderate, with just 52.4% of maternity units completing the survey. However, such response rates are common in survey data, and our response rate is similar to several other obstetric cross‐sectional surveys.^32^, ^33^, ^34^ Additionally, the sampling approach used was targeted to aim for consultant obstetricians with leadership and digital roles. The purpose was to retrieve responses from individuals who likely possess an in‐depth knowledge of current telehealth initiatives. However, survey completion is dependent on voluntary participation of the invited respondents, which might lead to selection bias. However, demographic comparisons between survey respondents and non‐respondents revealed no significant differences. Further, survey responses were based on self‐reported data, which is inherently subjective and represents a limitation common to survey‐based research. As the survey relied on a single respondent from each trust, the information provided might not have fully captured all local policies, guidelines, audits, safety evaluations, or outcome assessments. Consequently, findings regarding the availability of these processes should be interpreted as reported by respondents and were not independently verified at the institutional level. The survey was designed to encompass a broad range of questions, including both multiple choice and open‐ended questions, enabling a comprehensive assessment of remote monitoring and teleconsultation implementation. However, at times, the free‐text responses were brief, likely an inherent nature of a widely distributed survey to clinicians with busy schedules. Consequently, certain responses lacked clear context. Further, the maximal number of questions required to complete the survey was 52, which might have discouraged invitees from completing the survey. Finally, the survey was developed by a range of researchers with research background in digital technology initiatives. This ensured the proposed questions were of clinical utility and represents a strength of this study.

## 7 CONCLUSION

This survey study provides information regarding the current state of remote monitoring and obstetric teleconsultation implementation across the UK, primarily derived from English NHS trusts. In light of an evolving digital healthcare landscape, these technological strategies are of clinical and scientific relevance. Clear national frameworks are emerging and are vital to inform digital implementation and standardization across the nation. On an individual hospital level, continuous auditing and patient safety evaluation is paramount to ensure digital services uphold the same clinical standard as with face‐to‐face care. In an era of new innovative digital services, telemedicine strategies are an important avenue to explore, with the potential to modify current antenatal care models.

## AUTHOR CONTRIBUTIONS

J.L.V.: Conceptualization, data curation, formal analysis, funding acquisition, investigation, methodology, resources, writing – original draft and writing – review & editing. L.G.: Methodology, funding acquisition, supervision, validation and writing – review & editing. V.H.M.: Methodology, funding acquisition, supervision, validation and writing – review & editing. R.K.M.: Methodology, funding acquisition, supervision, validation and writing – review & editing.

## FUNDING INFORMATION

J. Le Vance and this study was funded by the Birmingham Women's and Children's Hospital Charity, grant number 2732009.

## CONFLICT OF INTEREST STATEMENT

The authors declare that they have no competing interests.

## Supporting information


**File S1.** Survey Questions.

**Table S1.** Checklist for Reporting Of Survey Studies (CROSS).

**Table S2:** Demographic characteristics of survey respondents versus non‐respondents.

**Table S3:** Results of survey respondents in maternity trusts which are previous users of remote monitoring.

## Data Availability

All data generated or analyzed during this study are included in this published article.

## References

[1] Le Vance J , Gurney L , Morris RK , Hodgetts Morton V . Telemedicine and remote monitoring advancements for obstetric antenatal care. Obstet Gynaecol Reprod. 2025;5:266‐270.

[2] Statistics OfN . Birth characteristics in England and Wales: 2021. 2023 https://www.ons.gov.uk/peoplepopulationandcommunity/birthsdeathsandmarriages/livebirths/bulletins/birthcharacteristicsinenglandandwales/2021

[3] Atkinson J , Hastie R , Walker S , Lindquist A , Tong S . Telehealth in antenatal care: recent insights and advances. BMC Med. 2023;21:332. 10.1186/s12916-023-03042-y PMC10470141 37649028

[4] Baudier P , Kondrateva G , Ammi C , Chang V , Schiavone F . Digital transformation of healthcare during the COVID‐19 pandemic: patients' teleconsultation acceptance and trusting beliefs. Technovation. 2023;120:102547.

[5] Sheikh A , Anderson M , Albala S , et al. Health information technology and digital innovation for national learning health and care systems. Lancet Digit Health. 2021;3:e383‐e396. 10.1016/S2589-7500(21)00005-4 33967002

[6] Chappell LC , Tucker KL , Galal U , et al. Effect of self‐monitoring of blood pressure on blood pressure control in pregnant individuals with chronic or gestational hypertension: the BUMP 2 randomized clinical trial. JAMA. 2022;327:1666‐1678. 10.1001/jama.2022.4726 PMC9066282 35503345

[7] Bekker MN , Koster MPH , Keusters WR , et al. Home telemonitoring versus hospital care in complicated pregnancies in The Netherlands: a randomised, controlled non‐inferiority trial (HoTeL). Lancet Digit Health. 2023;5:e116‐e124. 10.1016/S2589-7500(22)00231-X 36828605

[8] Palmer KR , Tanner M , Davies‐Tuck M , et al. Widespread implementation of a low‐cost telehealth service in the delivery of antenatal care during the COVID‐19 pandemic: an interrupted time‐series analysis. Lancet. 2021;398:41‐52. 10.1016/S0140-6736(21)00668-1 PMC8248925 34217399

[9] Le Vance J , Emms A , Hodgetts Morton V , Morris RK , Gurney L . Remote maternal‐fetal telemedicine monitoring for high‐risk pregnancy care: a feasibility study. PLoS One. 2025;20:e0336797. 10.1371/journal.pone.0336797 PMC12617905 41237146

[10] GOV.UK . Fit for the Future: 10 Year Health Plan for England ‐ Executive Summary (Accessible Version). 2025 https://www.gov.uk/government/publications/10‐year‐health‐plan‐for‐england‐fit‐for‐the‐future/fit‐for‐the‐future‐10‐year‐health‐plan‐for‐england‐executive‐summary

[11] NHS England . Better Births: Improving Outcomes of Maternity Services in England – A Five Year Forward View for Maternity Care. 2017 https://www.england.nhs.uk/publication/better‐births‐improving‐outcomes‐of‐maternity‐services‐in‐england‐a‐five‐year‐forward‐view‐for‐maternity‐care/

[12] NHS England . Maternity Transformation Programme. 2025 https://www.england.nhs.uk/mat‐transformation/

[13] NHS . Find Maternity Services. 2025 https://www.nhs.uk/service‐search/other‐health‐services/find‐maternity‐services

[14] Royal College of obstetricians and Gynecologists . Programme Team and Local Development Leads (LDLs). https://www.rcog.org.uk/about‐us/quality‐improvement‐clinical‐audit‐and‐research‐projects/each‐baby‐counts‐learn‐support/ebc‐ls‐programme‐overview/programme‐team‐and‐local‐development‐leads‐ldls/

[15] van den Heuvel JFM , Ayubi S , Franx A , Bekker MN . Home‐based monitoring and telemonitoring of complicated pregnancies: Nationwide cross‐sectional survey of current practice in The Netherlands. JMIR Mhealth Uhealth. 2020;8:e18966. 10.2196/18966 PMC7657725 33112250

[16] Jenn NC . Designing a questionnaire. Malays Fam Physician. 2006;1:32‐35. PMC4797036 26998209

[17] Sharma A , Minh Duc NT , Luu Lam Thang T , et al. A consensus‐based checklist for reporting of survey studies (CROSS). J Gen Intern Med. 2021;36:3179‐3187. 10.1007/s11606-021-06737-1 PMC8481359 33886027

[18] Mor L , Weiner E , Marom O , et al. The effect of home ultrasound on maternal anxiety in patients with previous recurrent pregnancy loss: a randomized control trial. Am J Obstet Gynecol MFM. 2024;6:101447. 10.1016/j.ajogmf.2024.101447 39122211

[19] Le Vance J , Adeoye A , Man R , et al. Remote home cardiotocography: a systematic review and meta‐analysis. PLoS Digit Health. 2026;5:e00011842. 10.1371/journal.pdig.0001184 PMC12795381 41525252

[20] Le Vance J , Vaughan M , Bhatia T , Gurney L , Hodgetts Morton V , Morris RK . Teleultrasound in obstetrics: a systematic review and meta‐analysis. PLoS Med. 2026;23:e1004922. 10.1371/journal.pmed.1004922 PMC12900445 41650130

[21] Le Vance J , Man R , Adeoye A , et al. Clinical outcome reporting in randomised controlled trials of remote obstetric antenatal monitoring: a systematic review. Eur J Obstet Gynecol Reprod Biol. 2026;322:115122. 10.1016/j.ejogrb.2026.115122 41996907

[22] Aasbø G , Blix E , Pay ASD , Waldum Å , Rivedal S , et al. Expectations related to home‐based telemonitoring of high‐risk pregnancies: a qualitative study addressing healthcare providers' and users' views in Norway. Acta Obstet Gynecol Scand. 2024;103:276‐285. 10.1111/aogs.14726 PMC10823400 37983832

[23] Bendix JM , Heinsen M , Backhausen MG . “But I'm not a professional”–how women with high‐risk pregnancies voice the experiences of home‐based telemonitoring; a qualitative interview study. Sex Reprod Healthc. 2024;41:101015. 10.1016/j.srhc.2024.101015 39180933

[24] NHS England . Inclusive Digital Healthcare: A Framework for NHS Action on Digital Inclusion. 2024 https://www.england.nhs.uk/long‐read/inclusive‐digital‐healthcare‐a‐framework‐for‐nhs‐action‐on‐digital‐inclusion/

[25] Ofcom . Adults' Media Use and Attitudes. 2025 https://www.ofcom.org.uk/media‐use‐and‐attitudes/media‐habits‐adults/adults‐media‐use‐and‐attitudes

[26] Good Things Foundation . Digital Inclusion in Health and Care. 2024 https://www.goodthingsfoundation.org/policy‐and‐research/research‐and‐evidence/research‐2024/health‐and‐care‐digital‐inclusion

[27] ACOG Committee Statement No. 20 . Ethical considerations with telehealth in obstetrics and gynecology. Obstet Gynecol. 2025;146:572‐582. 10.1097/AOG.0000000000006044 40966735

[28] Birnie E , Monincx WM , Zondervan HA , Bossuyt PM , Bonsel GJ . Cost‐minimization analysis of domiciliary antenatal fetal monitoring in high‐risk pregnancies. Obstet Gynecol. 1997;89:925‐929. 10.1016/s0029-7844(97)00150-6 9170466

[29] Carrandi A , Hu Y , Karger S , et al. Systematic review on the cost and cost‐effectiveness of mHealth interventions supporting women during pregnancy. Women Birth. 2023;36:3‐10. 10.1016/j.wombi.2022.03.007 35339412

[30] NHS . 2020/21 national tariff payment system. 2020 https://www.england.nhs.uk/wp‐content/uploads/2021/02/20‐21NT_Guidance_on_maternity_pathway_payment.pdf

[31] NHS England . Delivering a net zero NHS. 2025 https://www.england.nhs.uk/greenernhs/a‐net‐zero‐nhs/

[32] Grassl N , Nees J , Schramm K , et al. A web‐based survey assessing the attitudes of health care professionals in Germany toward the use of telemedicine in pregnancy monitoring: cross‐sectional study. JMIR Mhealth Uhealth. 2018;6:e10063. 10.2196/10063 PMC6105866 30089606

[33] Mallampati DP , Talati AN , Fitzhugh C , Enayet N , Vladutiu CJ , Menard MK . Statewide assessment of telehealth use for obstetrical care during the COVID‐19 pandemic. Am J Obstet Gynecol MFM. 2023;5:100941. 10.1016/j.ajogmf.2023.100941 PMC10035794 36965697

[34] Lanssens D , Vandenberk T , Lodewijckx J , et al. Midwives', obstetricians', and recently delivered mothers' perceptions of remote monitoring for prenatal care: retrospective survey. J Med Internet Res. 2019;21:e10887. 10.2196/10887 PMC6487343 30985286

