# Supplementary material for: Implementation of home‐based telemonitoring and teleconsultations for obstetrics: A cross‐sectional survey of current practice in the United Kingdom
Source: Int J Gynaecol Obstet. 2026 Jul 9;175(3):1649–63. doi: 10.1002/ijgo.71205 (PMC13769350; doi:10.1002/ijgo.71205)
Supplement: Supplementary file 1 [file IJGO-175-1649-s001.docx]

**Table S2:** Demographic characteristics of survey respondents versus non-respondents.

|  | | | **Respondents (n=76)** | **Percent (%) and 95% CI** | **Non-respondents (n=69)** | **Percent (%) and 95% CI** | **P value** |
| --- | --- | --- | --- | --- | --- | --- | --- |
| Nation | England | | 60/76 | 78.9 (68.1-87.5) | 57/69 | 82.6 (71.6-90.7) | 0.58 |
|  | Scotland | | 10/76 | 13.2 (6.5-22.9) | 7/69 | 10.1 (4.2-19.8) | 0.57 |
|  | Wales | | 3/76 | 3.9 (0.8-11.1) | 3/69 | 4.3 (0.9-12.2) | 1.00 |
|  | Northern Ireland | | 3/76 | 3.9 (0.8-11.1) | 2/69 | 2.9 (0.4-10.1) | 1.00 |
|  | | | | | | | |
| Births per year | | <1000 | 1/76 | 1.3 (0.0-7.1) | 4/69 | 5.8 (1.6-14.2) | 0.19 |
|  |  | 1000-2000 | 5/76 | 6.6 (2.2-14.7) | 6//69 | 8.7 (3.3 – 18.0) | 0.63 |
|  |  | 2001-3000 | 15/76 | 19.7 (11.5-30.5) | 9/69 | 13.0 (6.1-23.3) | 0.28 |
|  |  | 3001-4000 | 16/76 | 21.1 (12.5-31.9) | 13/69 | 18.8 (10.4-30.1) | 0.74 |
|  |  | 4001-5000 | 13/76 | 17.1 (9.4-27.5) | 18/69 | 26.1 (16.3-38.1) | 0.19 |
|  |  | 5001-6000 | 17/76 | 22.4 (13.6-33.4) | 8/69 | 11.6 (5.1-21.6) | 0.09 |
|  |  | 6001-7001 | 4/76 | 5.3 (1.5-12.9) | 3/69 | 4.3 (0.9-12.2) | 1.00 |
|  |  | 7001-8000 | 2/76 | 2.6 (0.3-9.2) | 4/69 | 5.8 (1.6-14.2) | 0,42 |
|  |  | >8001 | 3/76 | 3.9 (0.8-11.1) | 4/69 | 5.8 (1.6-14.2) | 0.71 |
|  | | | | | | | |
| Annual birth rate (median (range)) | | | 4300 (150 – 16200) | | 4130 (880-12300) | | 0.25 |
|  | | | | | | | |
| Level of maternity care | | District | 54/76 | 71.1 (59.5-80.9) | 46/69 | 66.7 (54.3-77.6) | 0.57 |
|  |  | Tertiary | 21/76 | 27.6 (18.0-39.1) | 18/69 | 26.1 (16.3-38.1) | 0.83 |
|  |  | Quaternary | 1/76 | 1.3 (0.0-7.1) | 5/69 | 7.2 (2.4-16.1) | 0.10 |
|  | | | | | | | |
| Teaching affiliated | | Yes | 64/76 | 84.2 (74.0-91.6) | 52/69 | 75.4 (63.5-84.9) | 0.18 |
|  |  | No | 12/76 | 15.8 (8.4-26.0) | 17/69 | 24.6 (15.1-36.5) |  |

CI, confidence interval; n,number
