# Supplementary material for: Implementation of home‐based telemonitoring and teleconsultations for obstetrics: A cross‐sectional survey of current practice in the United Kingdom
Source: Int J Gynaecol Obstet. 2026 Jul 9;175(3):1649–63. doi: 10.1002/ijgo.71205 (PMC13769350; doi:10.1002/ijgo.71205)
Supplement: Supplementary file 3 [file IJGO-175-1649-s003.docx]

**Table S3:** Results of survey respondents in maternity trusts which are previous users of remote monitoring.

|  | | **Frequency** | **Percent (%)** | **95% CI** |
| --- | --- | --- | --- | --- |
| **Reasons for discontinuation *** | Cost too high | 1/2 | 50.0 | 1.3-98.7 |
|  | Lack of clinical support | 1/2 | 50.0 | 1.3-98.7 |
|  | Lack of patient interest | 1/2 | 50.0 | 1.3-98.7 |
|  | | | | |
| **Type of home monitoring *** | Blood pressure monitoring | 2/2 | 100 | 15.8-100 |
|  | Glucose monitoring | 2/2 | 100 | 15.8-100 |
|  | Temperature measurement | 1/2 | 50.0 | 1.3-98.7 |
|  | Fetal electrocardiography | 1/2 | 50.0 | 1.3-98.7 |
|  | | | |  |
| **High-risk obstetric conditions using home monitoring *** | *Cardiovascular* | | |  |
|  | Chronic hypertension | 2/2 | 100 | 15.8-100 |
|  | Gestational hypertension | 2/2 | 100 | 15.8-100 |
|  | Pre-eclampsia | 1/2 | 50.0 | 1.3-98.7 |
|  | *Endocrine and metabolic* | | |  |
|  | Gestational diabetes | 2/2 | 100 | 15.8-100 |
|  | *Hepatobiliary* | | | |
|  | Obstetric cholestasis | 1/2 | 50.0 | 1.3-98.7 |
|  | | | |  |
| **Healthcare professional reviewing home monitoring data *** | Obstetric consultant | 2/2 | 100 | 15.8-100 |
|  | Midwife | 2/2 | 100 | 15.8-100 |
|  | Specialist midwife | 1/2 | 50.0 | 1.3-98.7 |
|  | Doctor – non consultant | 1/2 | 50.0 | 1.3-98.7 |
|  | | | |  |
| **Method of communication to feedback results to patient *** | Phone call | 2/2 | 100 | 15.8-100 |
|  | Video call | 1/2 | 50.0 | 1.3-98.7 |
|  | | | |  |
| **Barriers for home monitoring *** | Non-compliance issues | 2/2 | 100 | 15.8-100 |
|  | Language barriers | 1/2 | 50.0 | 1.3-98.7 |
|  | | | |  |
| **Minimum gestational age for home monitoring** | First trimester | 0/2 | 0.0 | 0.0-84.2 |
|  | Second trimester | 0/2 | 0.0 | 0.0-84.2 |
|  | Third trimester | 0/2 | 0.0 | 0.0-84.2 |
|  | Unsure | 0/2 | 0.0 | 0.0-84.2 |
|  | No minimum gestation | 2/2 | 100 | 15.8-100 |
|  | | | |  |
| **Local guideline for home monitoring** | Yes | 1/2 | 50.0 | 1.3-98.7 |
|  | No | 0/2 | 0.0 | 0.0-84.2 |
|  | Not sure | 1/2 | 50.0 | 1.3-98.7 |
|  | | | |  |
| **Patient safety evaluation prior to using home monitoring** | Yes | 1/2 | 50.0 | 1.3-98.7 |
|  | No | 0/2 | 0.0 | 0.0-84.2 |
|  | Not sure | 1/2 | 50.0 | 1.3-98.7 |
|  | | | |  |
| **Assessment of home monitoring on patient experiences** | Yes | 1/2 | 50.0 | 1.3-98.7 |
|  | No | 1/2 | 50.0 | 1.3-98.7 |
|  | Not sure | 0/2 | 0.0 | 0.0-84.2 |
|  | | | |  |
| **Assessment of home monitoring on clinical outcomes** | Yes | 1/2 | 50.0 | 1.3-98.7 |
|  | No | 0/2 | 0.0 | 0.0-84.2 |
|  | Not sure | 1/2 | 50.0 | 1.3-98.7 |
|  | | | |  |
| **Assessment of home monitoring on healthcare service outcomes** | Yes | 1/2 | 50.0 | 1.3-98.7 |
|  | No | 0/2 | 0.0 | 0.0-84.2 |
|  | Not sure | 1/2 | 50.0 | 1.3-98.7 |

CI ,confidence interval

*Respondents were able to select multiple answers
