# Supplementary material for: Implementation of home‐based telemonitoring and teleconsultations for obstetrics: A cross‐sectional survey of current practice in the United Kingdom
Source: Int J Gynaecol Obstet. 2026 Jul 9;175(3):1649–63. doi: 10.1002/ijgo.71205 (PMC13769350; doi:10.1002/ijgo.71205)
Supplement: Supplementary file 4 [file IJGO-175-1649-s004.docx]

Survey Questions

**Section 1: General questions**

1. What is the name of the hospital trust you are currently working at?
   a. Open question.
2. What is the average number of births per year at your hospital?
   a. Open question.
3. What best describes your hospital:
   a. District hospital
   b. Tertiary hospital
   c. Quaternary hospital
4. Is your hospital a teaching hospital?
   a. Yes
   b. No

**Section 2: Home based antenatal monitoring in obstetrics.**

1. Does your hospital currently offer home-based monitoring in your hospital? (The patient completed antenatal monitoring at home by themselves)
   a. Yes (to complete section 2.1)
   b. No, but have in the past (to complete section 2.2)
   c. No (to complete section 2.3)

**Section 2.1: Home- based monitoring questions (if you ticked that your hospital currently has home-based monitoring)**

1. What types of home-based monitoring do you offer?
   a. Self-measured symphysial fundal height
   b. Blood glucose monitoring
   c. Urine testing
   d. Blood pressure measurement
   e. Temperature measurement
   f. Blood testing
   g. Maternal heart rate measurement
   h. Fetal handheld Doppler
   i. Home CTG
   j. Home fetal ECG
   k. Home ultrasound
   l. Other:
2. If you ticked ‘other’ on the previous question, please provide the types of home monitoring below.
   a. Open question.
3. To the best of your knowledge, what year did your hospital first offer home-based patient monitoring?
   a. Open question
4. Do you offer home-based monitoring to low or high-risk obstetric patients?
   a. Low risk
   b. High risk
   c. Both
5. If you offer it to high-risk patients, which high-risk conditions does this include?
   a. Fetal growth restriction
   b. Preterm prelabour rupture of membrane
   c. Pre-eclampsia
   d. Gestational hypertension
   e. Chronic hypertension
   f. Decreased fetal movements
   g. Obstetric cholestasis
   h. Gestational diabetes mellitus
   i. Prolonged prelabour rupture of membranes at term
   j. Oligohydramnios
   k. Polyhydramnios
   l. Multiple pregnancy
   m. Fetal anomalies
   n. Social or psychological reasons
   o. Poor obstetric history
   p. Other:
6. If you ticked ‘other’ on the previous question, please provide the high-risk conditions below.
   a. Open question.
7. Does your hospital review the home-based monitoring data remotely?
   a. Yes
   b. No
   c. Not sure
8. If yes to question 5, is the home monitoring reviewed in real time? If use multiple forms of home-based monitoring, please specify which are reviewed in real time.
   a. Open question.
9. Who is responsible for reviewing the home-based monitoring data?
   a. Healthcare assistant
   b. Midwife
   c. Specialist midwife
   d. Doctor – non consultant
   e. Consultant
   f. Other
10. If you ticked ‘other’ on the previous question, please provide the person responsible for reviewing the home-based monitoring data.
    a. Open question.
11. How are the home monitoring results and interpretation relayed back to the patient?
    a. Text message
    b. Phone call
    c. Video call
    d. Via patient portals
    e. Other
12. If you ticked ‘other’ o the previous question, please write how the home monitoring results are relayed back to the patient.
    a. Open question.
13. Does your hospital have a local guideline for home-based pregnancy monitoring?
    a. Yes
    b. No
    c. Not sure
14. Does your hospital conduct a patient safety evaluation prior to the usage of home-based monitoring technology?
    a. Yes
    b. No
    c. Not sure
15. Does your hospital assess patient experiences of home-based monitoring following implementation?
    a. Yes
    b. No
    c. Not sure
16. Does your hospital assess the impact of home-based monitoring on clinical outcomes (maternal and/or perinatal) ? If so, which outcomes?
    a. Open question
17. Does your hospital assess the impact of home-based monitoring on service outcomes (e.g. admission, patient satisfaction, cost, attendance to maternity triage)? If so, which outcomes?
    a. Open question
18. Does your hospital have any contraindications when offering patients home-based monitoring?
    a. Distance of patient’s home to the hospital
    b. Non-compliance issues
    c. Language barriers
    d. Substance misuse
    e. Certain clinical reason
    f. Other
19. If you chose a clinical reason or other to the previous question, please detail the specifics below.
    a. Open question.
20. Is there a minimum gestational age which is eligible for home-based monitoring?
    a. Open question.
21. Have you noticed an increase in the number of patients using home-based monitoring following the pandemic?
    a. Yes
    b. No
    c. Not sure

**Section 2.2: Home-based monitoring questions (if you ticket that your hospital had any type of home-based monitoring but now it has been discontinued)**

1. What types of home-based monitoring did you offer?
   a. Self-measured symphysial fundal height
   b. Blood glucose monitoring
   c. Urine testing
   d. Blood pressure measurement
   e. Temperature measurement
   f. Home blood testing
   g. Maternal heart rate measurement
   h. Fetal handheld Doppler
   i. Home cardiotocography
   j. Home ultrasound
   k. Other:
2. If you ticked ‘other’ on the previous question, please provide the reasons why home-based monitoring was discontinued.
   a. Open question.
3. To the best of your knowledge, which years did your hospital provide home-based monitoring?
   a. Open question
4. What were the reasons why your hospital discontinued home-based monitoring?
   a. Cost to high
   b. Not enough patients using the service
   c. Not enough support from the clinical team
   d. Not enough interest from patients
   e. Not enough clinical capacity
   f. Adverse outcome/s occurred leading to service discontinuing
   g. Other:
5. If you ticked ‘other’ on the previous question, please provide the types of home monitoring below.
   a. Open question.
6. Did you offer home-based telemonitoring to low or high-risk obstetric patients?
   a. Low risk
   b. High risk
   c. Both
7. If you offered it to high-risk patients, which high-risk conditions did this include?
   a. Fetal growth restriction
   b. Preterm prelabour rupture of membrane
   c. Pre-eclampsia
   d. Gestational hypertension
   e. Chronic hypertension
   f. Decreased fetal movements
   g. Obstetric cholestasis
   h. Gestational diabetes mellitus
   i. Prolonged prelabour rupture of membranes at term
   j. Oligohydramnios
   k. Polyhydramnios
   l. Multiple pregnancy
   m. Fetal anomalies
   n. Social or psychological reasons
   o. Poor obstetric history
   p. Other:
8. If you ticked ‘other’ on the previous question, please provide the types of high-risk conditions you would have offered home-based monitoring to.
   a. Open question.
9. Was the home-based monitoring data reviewed remotely by your hospital?
   d. Yes
   e. No
   f. Not sure
10. If yes, was it reviewed in real time? If used multiple forms of home-based monitoring, please specify which were reviewed in real time.
    a. Open question.
11. Who was responsible for reviewing the home-based monitoring data?
    a. Healthcare assistant
    b. Midwife
    c. Specialist midwife
    d. Doctor – non consultant grade
    e. Consultant
    f. Other
12. If you ticked ‘other’ on the previous question, please provide the people who were responsible for review the home-based monitoring data.
    a. Open question.
13. If yes, how were the results and interpretation relayed back to the patient?
    a. Text message
    b. Phone call
    c. Video call
    d. Via patient portals
    e. Other
14. If you ticked ‘other’ on the previous question, please provide how home monitoring results were relayed back to the patient.
    a. Open question.
15. Did your hospital have a local guideline for home-based pregnancy monitoring?
    a. Yes
    b. No
    c. Not sure
16. Did your hospital conduct a patient safety evaluation prior to the usage of home-based monitoring technology?
    a. Yes
    b. No
    c. Not sure
17. Did your hospital assess patient experiences of home-based monitoring following implementation?
    a. Yes
    b. No
    c. Not sure
18. Did your hospital assess the impact of home-based monitoring on clinical outcomes? If so, which outcomes?
    a. Open question
19. Did your hospital assess the impact of home-based monitoring on service outcomes (e.g. admission, patient satisfaction, cost, attendance to maternity triage)? If so, which outcomes?
    a. Open question
20. Did your hospital have any contraindications when offering patients home-based monitoring?
    a. Distance of patient’s home to the hospital
    b. Non-compliance issues
    c. Language barriers
    d. Substance misuse
    e. Clinical reason (if so, specify):
    f. Other
21. If you ticked ‘other’ on the previous question, please provide how home monitoring results were relayed back to the patient.
    a. Open question.
22. was there a minimum gestational age which is eligible for home-based monitoring?
    a. Open question:
23. Is your hospital planning to recommence home-based monitoring?
    a. Yes
    b. No

**Section 2.3: Home monitoring questions (if you ticked that your hospital has never had home-based monitoring)**

1. Why is your hospital not providing home-based monitoring?
   a. Cost to high
   b. Not enough potential patients to use the service
   c. Not enough support from the clinical team
   d. Not enough interest from patients
   e. Not enough clinical capacity
   f. Other:
2. If you ticked ‘other’ on the previous question, please state the reasons below.
   a. Open question.
3. Is your hospital considering the provision of home-based monitoring in the future? If yes, what types of home monitoring and why? If no, why?
   a. Open question.

**Section 3: Tele consultations in obstetrics**

2. Does your hospital currently conduct tele-consultations? (virtual consultations between provider and another person, which can either be a patient or a different specialist)
   a. Yes (to complete section 3.1)
   b. No, but have in the past (to complete section 3.2)
   c. No (to complete section 3.3)

**Section 3.1: Tele-consultations questions (only complete if your hospital currently have it)**

1. To the best of your knowledge, when did your hospital first offer tele-consultations services?
   a. Open question
2. What does your hospital use tele-consultation services for?
   a. Pre-pregnancy patient appointments
   b. Antenatal clinic patient appointments
   c. To regularly discuss with other specialists (during the antenatal period)
   d. To discuss/review home-based antenatal monitoring results with patients
   e. Antenatal tele-ultrasound services (transfer of ultrasound data either in real-time or delayed) with remote consultation with clinician or patient.
   f. Other
3. If you ticked ‘other’ on the previous question, please provide the reasons you use teleconsultations in obstetrics.
   a. Open question.
4. How do you conduct tele-consultations with patients?
   a. Phone call
   b. Video call
   c. Text message
   d. Via patient portals
   e. Other
5. If you ticked ‘other’ on the previous question, please provide how you conduct teleconsultations with patients in obstetrics.
   a. Open question.
6. Who is responsible for conducting the tele-consultations at your hospital?
   a. Midwife
   b. Specialist midwife
   c. Doctor – non consultant
   d. Consultant
   e. Other
7. If you ticked ‘other’ on the previous question, please provide who is responsible for conducting teleconsultations.
   a. Open question.
8. Do you offer tele-consultations to low or high-risk obstetric patients?
   a. Low risk
   b. High risk
   c. Both
9. If you offer it to high-risk patients, which high-risk conditions does this include?
   a. Fetal growth restriction
   b. Preterm prelabour rupture of membrane
   c. Pre-eclampsia
   d. Gestational hypertension
   e. Chronic hypertension
   f. Decreased fetal movements
   g. Obstetric cholestasis
   h. Gestational diabetes mellitus
   i. Prolonged prelabour rupture of membranes at term
   j. Oligohydramnios
   k. Polyhydramnios
   l. Multiple pregnancy
   m. Fetal anomalies
   n. Social or psychological reasons
   o. Poor obstetric history
   p. Other
10. If you ticked ‘other’ on the previous question, please provide which high-risk conditions you offer teleconsultations to.
    a. Open question.
11. Does your hospital have a local guideline for conducting obstetric tele-consultations?
    a. Yes
    b. No
    c. Not sure
12. Does your hospital conduct a patient safety evaluation prior to the usage of obstetric tele-consultations?
    a. Yes
    b. No
    c. Not sure
13. Does your hospital assess patient experiences of obstetric tele-consultations following implementation?
    a. Yes
    b. No
    c. Not sure
14. Does your hospital assess the impact of obstetric tele-consultations on clinical outcomes? If so, which outcomes?
    a. Open question
15. Does your hospital assess the impact of obstetric tele-consultations on service outcomes (e.g. admission, patient satisfaction, cost, attendance to maternity triage)? If so, which outcomes?
    a. Open question
16. Does your hospital have any contraindications when offering obstetric patients tele-consultations?
    a. Distance of patient’s home to the hospital
    b. Non-compliance issues
    c. Language barriers
    d. Substance misuse
    e. Clinical reason (if so, specify):
    f. Other
17. If you ticked ‘other’ on the previous question, please detail the specifics below.
    a. Open question.
18. Is there a minimum gestational age which is eligible for tele-consultations during the antenatal period?
    a. Open question:
19. Has there been an increase in the number of patients using tele-consultations following the pandemic?
    a. Yes
    b. No
    c. Not sure

**Section 3.2: Tele-consultation questions (only complete if your hospital had tele-consultations but then discontinued it)**

1. What were the reasons why your hospital discontinued tele-consultations?
   a. Cost to high
   b. Not enough patients using the service
   c. Not enough support from the clinical team
   d. Not enough interest from patients
   e. Not enough clinical capacity
   f. Adverse outcome/s occurred leading to the service discontinuing
   g. Other
2. If you ticked ‘other’ on the previous question, please provide the reasons why teleconsultations were discontinued.
   a. Open question.
3. What did your hospital use tele-consultation services for?
   a. Pre-pregnancy patient appointments
   b. Antenatal clinic patient appointments
   c. To regularly discuss with other specialists (during the antenatal period)
   d. To discuss/review home-based antenatal monitoring results with patients
   e. Antenatal tele-ultrasound services (transfer of ultrasound data either in real-time or delayed) with remote consultation with clinician or patient.
   f. Other
4. If you ticked ‘other’ on the previous question, please provide what your hospital used teleconsultations for.
   a. Open question.
5. To the best of your knowledge, from which years did your hospital provide tele-consultations?
   a. Open question
6. How did your hospital conduct your tele-consultations with patients?
   a. Phone call
   b. Video call
   c. Text message
   d. Via patient portals
   e. Other
7. If you ticked ‘other’ on the previous question, please provide how teleconsultations were conducted with patients.
   a. Open question.
8. Who was responsible for conducting the tele-consultations at your hospital?
   a. Midwife
   b. Specialist midwife
   c. Doctor – non consultant
   d. Consultant
   e. Other
9. If you ticked ‘other’ on the previous question, please provide who was responsible for conducting teleconsultations.
   a. Open question.
10. Did you offer tele-consultations to low or high-risk obstetric patients?
    a. Low risk
    b. High risk
    c. Both
11. If you offered it to high-risk patients, which high-risk conditions did this include?
    a. Fetal growth restriction
    b. Preterm prelabour rupture of membrane
    c. Pre-eclampsia
    d. Gestational hypertension
    e. Chronic hypertension
    f. Decreased fetal movements
    g. Obstetric cholestasis
    h. Gestational diabetes mellitus
    i. Prolonged prelabour rupture of membranes at term
    j. Oligohydramnios
    k. Polyhydramnios
    l. Multiple pregnancy
    m. Fetal anomalies
    n. Social or psychological reasons
    o. Poor obstetric history
    p. Other
12. If you ticked ‘other’ on the previous question, please provide the types of high-risk conditions teleconsultations were provided to.
    a. Open question.
13. Did your hospital have a local guideline for conducting obstetric tele-consultations?
    a. Yes
    b. No
    c. Not sure
14. Did your hospital conduct a patient safety evaluation prior to the usage of obstetric tele-consultations?
    a. Yes
    b. No
    c. Not sure
15. Did your hospital assess patient experiences of obstetric tele-consultations following implementation?
    a. Yes
    b. No
    c. Not sure
16. Did your hospital assess the impact of tele-consultations on clinical outcomes? If so, which outcomes?
    a. Open question
17. Did your hospital assess the impact of tele-consultations on service outcomes (e.g. admission, patient satisfaction, cost, attendance to maternity triage)? If so, which outcomes?
    a. Open question
18. Did your hospital have any contraindications when offering patients tele-consultations?
    a. Distance of patient’s home to the hospital
    b. Non-compliance issues
    c. Language barriers
    d. Substance misuse
    e. Clinical reason (if so, specify):
    f. Other
19. If you chose a clincal reason or other to the previous question, please detail the specifics below.
    a. Open question.
20. Was there a minimum gestational age which is eligible for tele-consultations during the antenatal period?
    a. Open question.
21. Is your hospital planning to recommence tele-consultations?
    a. Yes
    b. No

**Section 3.3: Tele-consultations questions (if you ticked that your hospital never had teleconsultations)**

1. Why is your hospital not providing tele-consultations?
   b. Cost to high
   c. Not enough potential patients to use the service
   d. Not enough support from the clinical team
   e. Not enough interest from patients
   f. Not enough clinical capacity
   g. Other
2. If you ticked ‘other’ on the previous question, please provide the types of high-risk conditions teleconsultations were provided to.
   a. Open question.
3. Is your hospital considering the provision of teleconsultations? If yes, what types of teleconsultations and why? If no, why?
   a. Open question.

**Section 4: All hospitals – general questions.**

1. What are your three main advantages for home-based monitoring?
   a. Open question.
2. What are your three main disadvantages for home-based monitoring?
   a. Open question.
3. What are your three main advantages for tele-consultations?
   a. Open question.
4. What are your three main disadvantages for tele-consultations?
   a. Open question.
